# Whole-Genome Sequences of 13 Endophytic Bacteria Isolated from Shrub Willow (*Salix*) Grown in Geneva, New York

**DOI:** 10.1128/genomeA.00288-14

**Published:** 2014-05-08

**Authors:** Huan You Gan, Han Ming Gan, Michael A. Savka, Alexander J. Triassi, Matthew S. Wheatley, Lawrence B. Smart, Eric S. Fabio, André O. Hudson

**Affiliations:** a School of Science, Monash University Malaysia, Jalan Lagoon Selatan, Bandar Sunway, Selangor, Malaysia; b The Thomas H. Gosnell School of Life Sciences, Rochester Institute of Technology, Rochester, New York, USA; c Department of Horticulture, Cornell University, New York State Agricultural Experiment Station, Geneva, New York, USA

## Abstract

Shrub willow, *Salix* spp. and hybrids, is an important bioenergy crop. Here we report the whole-genome sequences and annotation of 13 endophytic bacteria from stem tissues of *Salix purpurea* grown in nature and from commercial cultivars and *Salix viminalis* × *Salix miyabeana* grown in bioenergy fields in Geneva, New York.

## GENOME ANNOUNCEMENT

Bioenergy produced from plant biomass as a feedstock has the potential to mitigate concerns regarding climate change and sustainability, among others (1). Shrub willow, *Salix* spp. and hybrids, is an attractive plant for bioenergy given its rapid growth and sustainable growth characteristics (2). There have been numerous efforts regarding the development of shrub willow cultivars suitable for the production of bioenergy (3). Endophytes have been isolated from tissues of tree willows in nature (4), but it is unknown what role endophytes might play in shrub willows growing in intensively managed bioenergy fields. To gain some insights into endophytic bacteria that associate with shrub willow, we embarked on a project which resulted in the identification of 69 unique bacteria, of which 13 were subjected to whole-genome sequencing and annotation. Plant-associated bacteria that are beneficial to *Salix* have the potential to improve crop production while reducing need for inputs. In addition, the identification of bacteria that are detrimental to growth and development through phytopathogenesis is also of interest as a foundation for breeding for resistance and maximizing growth potential. The endophytic bacteria were initially isolated from surface-sterilized stem tissues from wild accessions of *S. purpurea* growing in nature and from commercial cultivars of *S. purpurea* and *S. viminalis* × *S. miyabeana* growing in fertilized and nonfertilized bioenergy fields in Geneva, NY. The 13 endophytes were initially identified by amplification and nucleotide sequence analysis of the variable 3 region of the 16S rRNA gene (5).

Genomic DNA was isolated from the endophytes using a GenElute bacterial genomic kit (Sigma-Aldrich, St. Louis, MO) and prepared for whole-genome sequencing using a Nextera XT library preparation kit (Illumina, San Diego, CA). Whole-genome sequencing was performed using the Illumina Miseq (150-bp paired-end reads). The reads were error corrected and assembled *de novo* using Spades 2.5 (6). Scaffolding of the contigs and *in silico* gap-closing of the resulting scaffolds were performed with SSPACE and GapFiller, respectively (7, 8). Genome annotation was performed using the Prokka annotation pipeline, which incorporated Prodigal 2.60, Aragorn, and RNAmmer 1.2 for the prediction of open reading frames (ORFs), tRNAs, and rRNAs, respectively (9–11). Additional annotation of the predicted protein sequences was done using InterProScan5 (12). The key attributes for the genome sequences and annotation are summarized in Table 1. An in-depth analysis of the genes associated with plant-microbe symbiosis is under way and will be published in a subsequent report.

**TABLE 1:** Sequencing and annotation results for the 13 endophytes isolated from *Salix*

Strain	Source^*a*^	SubID	BioProject no.	BioSample no.	Accession no.	Organism	Genome coverage (×)	Genome size (bp)	No. of contigs	No. of ORFs	No. of tRNAs	No. of rRNAs
RIT273	Fabius	SUB467427	PRJNA239282	SAMN02676620	JFOK00000000	*Pantoea agglomerans*	138	5,365,338	26	4,914	75	17
RIT283	Fabius	SUB467430	PRJNA239283	SAMN02676621	JFOJ00000000	*Staphylococcus haemolyticus*	289	2,527,922	81	2,445	49	14
RIT288	Fish Creek	SUB468060	PRJNA239284	SAMN02676622	JFYN00000000	*Pseudomonas* sp.	191	6,273,290	44	5,547	61	10
RIT293	Fabius	SUB468074	PRJNA239285	SAMN02676623	JFYO00000000	*Microbacterium oleivorans*	194	2,898,622	11	2,782	49	8
RIT304	Wild	SUB468076	PRJNA239286	SAMN02676624	JFYP00000000	*Micrococcus luteus*	166	2,506,829	183	2,248	53	4
RIT305	Wild	SUB468079	PRJNA239287	SAMN02676625	JFYQ00000000	*Micrococcus luteus*	200	2,612,381	110	2,350	49	6
RIT308	Fabius	SUB468082	PRJNA239288	SAMN02676626	JFYR00000000	*Janthinobacterium lividum*	189	6,212,741	44	5,431	83	19
RIT309	Fabius	SUB468084	PRJNA239289	SAMN02676627	JFYS00000000	*Stenotrophomonas* sp.	194	4,634,795	45	4,141	77	6
RIT313	Fabius	SUB468085	PRJNA239290	SAMN02676628	JFYT00000000	*Delftia* sp.	56	6,698,360	122	5,936	78	5
RIT324w	Fabius	SUB468086	PRJNA239291	SAMN02676629	JFYU00000000	*Micrococcus luteus*	459	2,635,230	118	2,381	53	7
RIT328	Fish Creek	SUB468089	PRJNA239292	SAMN02676630	JFYV00000000	*Sphingomonas* sp.	73	4,343,511	56	4,002	58	3
RIT341	Fabius	SUB468091	PRJNA239293	SAMN02676631	JFYW00000000	*Exiguobacterium* sp.	138	3,107,022	15	3,168	62	20
RIT357	Wild	SUB468093	PRJNA239294	SAMN02676632	JFYX00000000	*Pseudomonas* sp.	69	3,107,022	49	5,552	60	7

a Wild, wild *Salix purpurea*; Fish Creek, *Salix purpurea* Fish Creek cultivar; Fabius, *Salix viminalis* × *S. miyabeana* Fabius cultivar.

### Nucleotide sequence accession numbers.

The nucleotide sequences have been deposited at DDBJ/EMBL/GenBank under the accessions numbers provided in Table 1.
